# Extracorporeal Photopheresis in Children with Chronic Graft-Versus-Host Disease

**DOI:** 10.3390/ph14080808

**Published:** 2021-08-17

**Authors:** Andrey Kozlov, Maria Estrina, Olesia Paina, Tatiana Bykova, Anna Osipova, Polina Kozhokar, Zhemal Rakhmanova, Irina Solodova, Elena Morozova, Alexander Alyansky, Irina Kulagina, Asmik Gevorgian, Anna Dotsenko, Ivan Moiseev, Alexey Chukhlovin, Alexander Kulagin, Sergey Bondarenko, Elena Semenova, Ludmila Zubarovskaya

**Affiliations:** RM Gorbacheva Research Institute, Pavlov University, 197022 St. Petersburg, Russia; estrina.ma@mail.ru (M.E.); paina@mail.ru (O.P.); dr.bykova@mail.ru (T.B.); md.annarats@gmail.com (A.O.); kozhokar.polina@gmail.com (P.K.); rakhmanovazhemal@gmail.com (Z.R.); sirp@bk.ru (I.S.); dr_morozova@mail.ru (E.M.); aalyanskiy@gmail.com (A.A.); kulagina1965@inbox.ru (I.K.); asmikgevorgian@gmail.com (A.G.); aa.dotcenko@gmail.com (A.D.); moisiv@mail.ru (I.M.); alexei.chukh@mail.ru (A.C.); kulagingem@rambler.ru (A.K.); dr.sergeybondarenko@gmail.com (S.B.); alena-semenova@yandex.ru (E.S.); zubarovskaya_ls@mail.ru (L.Z.)

**Keywords:** children, chronic graft versus host disease, extracorporeal photopheresis

## Abstract

Chronic graft versus host disease (cGVHD) remains a major complication of allogeneic hematopoietic stem cell transplantation (allo-HSCT). It significantly decreases survival and quality of life. The present study demonstrates retrospective data on extracorporeal photopheresis (ECP) in children with cGVHD. A total of 42 children with steroid-refractory cGVHD were enrolled in the study. The majority of patients had acute leukemia (*n* = 32, 76%). All patients received ECP as second (*n* = 18, 43%) or third (*n* = 24, 57%) line of therapy. Initial ECP schedule consisted of bimonthly regimen for two consecutive days with possibility of further tapering according to response. Any concurrent treatment administered before ECP could be continued if considered necessary. Complete response to ECP was registered in seven (17%) patients and partial response in 24 (57%). Overall response according to organ involvement was as follows: skin (*n* = 24, 75%), mucous membranes (*n* = 16, 73%), liver (*n* = 8, 80%), gut (*n* = 4, 80%), lungs (*n* = 2, 22%) and joints (*n* = 2, 67%). Five-year overall, progression-free and failure-free survival was 57%, 56% and 30%, respectively. Non-relapse mortality at 5 years was 14%. We didn’t observe any clinically significant complications in children that could be attributed to the procedure. ECP remains important and safe treatment option in children with cGVHD.

## 1. Introduction

Chronic graft-versus-host disease (cGVHD) remains a major complication of allogeneic hematopoietic stem cell transplantation (allo-HSCT). This disorder may significantly decrease survival and quality of life, despite efficient preventive and therapeutic options. Non-relapse mortality (NRM) in surviving patients with cGVHD is increased within decades after allo-HSCT [1]. More recently, posttransplant cyclophosphamide (PtCy) and ruxolitinib became game changers that improved clinical outcome [2,3,4,5]. These innovative approaches were readily implemented into routine practice, thus resulting in lower cGVHD incidence and excellent control of cGVHD symptoms in most patients. Autoimmune-like symptoms are characteristic to cGVHD, suggesting a multimodal pathogenesis which is currently poorly understood, with immune dysregulation being the leading mechanism [6,7,8]. Resulting immune-mediated inflammatory reactions and connective tissue fibrosis comprise the pathophysiology of cGVHD [5]. 

Treatment of cGVHDutilizes immunosupressive and/or immunomodulatory therapies. It is always a challenge to treat a disorder with unclear and complex pathogenesis since exact mechanisms of distinct curative approaches are not well defined. With emergence of new treatment options, the role of previously established therapies in children should be reassessed, including extracorporeal photopheresis (ECP). Precise biological action of ECP is unknown, but apoptosis of pathogenic T-cells is supposed to be the most significant factor. Tolerogenic dendritic cells cause switch of immune response profile, whereas increase in the number of T-regulatory cells and natural killer cells are also probable effects of ECP [9]. 

Existing information on ECP usage in pediatrics is limited to pilot studies. But, as in other disorders, cGVHD may have specific pediatric features compared to adults, with distinct patterns of clinical response. These circumstances necessitate analysis of ECP effectiveness in children separately from adults. ECP is used for cGVHD treatment for approximately 25 years [10]. Multiple pilot studies demonstrated its efficiency in steroid-refractory (SR)cGVHD [11]. However, only few randomized trials were performed, with major limitations for the study design, thus precluding definite evaluation of ECP effects in cGVHD management [12,13]. The aim of present study was to summarize retrospective data from our clinics on ECP efficiency in children with SR cGVHD.

## 2. Results

Patients characteristics are listed in Table 1.

Complete response to ECP-based therapy was registered in seven (17%) patients and partial response in 24 (57%). Overall response (ORR) according to organ involvement was as follows: skin (*n* = 24, 75%), mucous membranes (*n* = 16, 73%), liver (*n* = 8, 80%), gut (*n* = 4, 80%), lungs (*n* = 2, 22%) and joints (*n* = 2, 67%) (Table 2).

**Figure 1:**
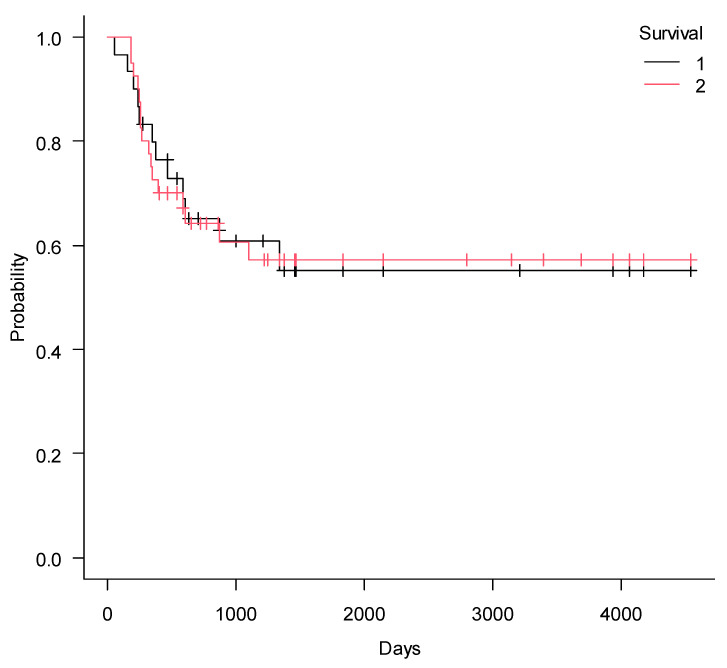
Progression-free survival (1) and overall survival (2) in children with cGVHD after ECP.

**Figure 2:**
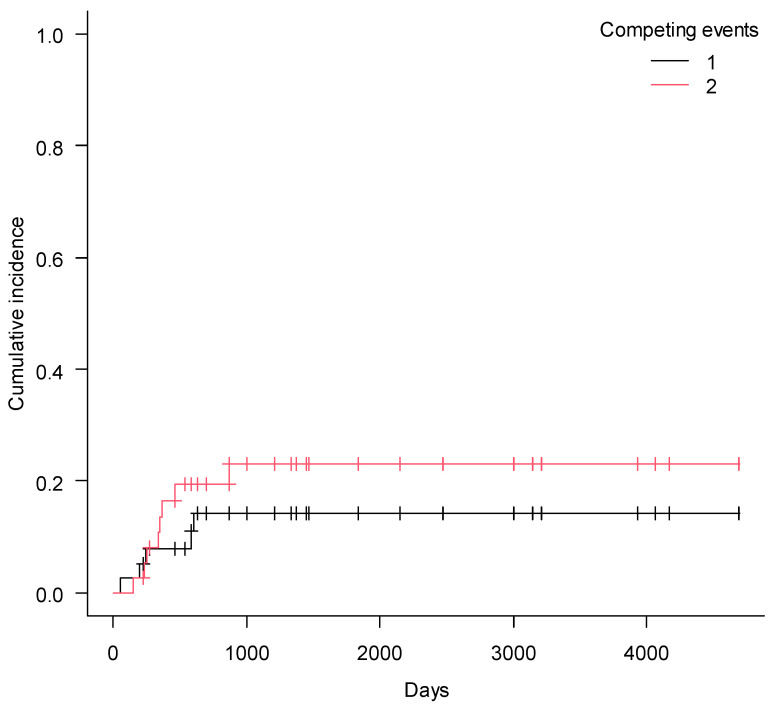
Non-relapse mortality (1) and cumulative incidence of relapse (2) in children with cGVHD after ECP.

**Figure 3:**
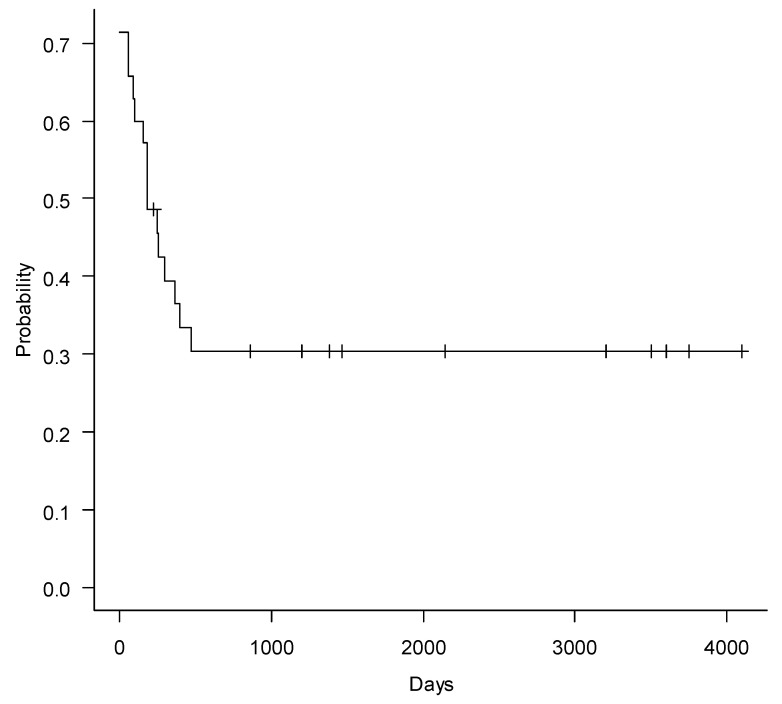
Failure-free survival in children with cGVHD after ECP.

Prior to ECP administration median dosage of steroids was 0.5 mg/kg/day (range 0–1) and after treatment with ECP median dosage was 0.25 mg/kg/day (range 0–2) (*p* = 0.0003). Overall steroids were tapered in 25 children (66%). Non-relapse mortality (NRM) after 5 years of observation was 14% (CI95%, 5–28%) and cumulative incidence of relapse (CIR), 23% (CI95%, 11–38%).Relapse of the malignancy (*n* = 11) was the main cause of death, and only four patients succumbed to cGVHD. With median follow-up of 774 days (180–4541), OS and PFS at 5 years were 57% (CI95%, 39–72%) and 56% (CI95%, 37–72%), respectively. FFS at 5 years was 30% (CI95% 16–46%) (Figure 1, Figure 2 and Figure 3).

Eighteen patients (43%) required correction of IST after ECP treatment, due to insufficient clinical effect. Only response to ECP was associated with improved OS (*p* = 0.0002). No other potential prognostic factors affected survival in the study (Table 3). Multivariate analysis was not performed as only one factor was statistically significantly associated with survival.

We didn’t observe any clinically significant complications attributable to ECP. The most common adverse effects were catheter-associated (*n* = 5) which did not result into the ECP discontinuation. Three patients had recurrent venous thrombosis, and catheter-associated infections were detected in two cases. One patient developed a severe blood electrolyte imbalance that was successfully corrected. Among eight children that received combination of ECP with ruxolitinib one episode (13%) of transient cytopenia was registered. It did not result in discontinuation or correction of therapy. Other well-known complications of ECP, such as arterial hypotension, decrease in peripheral blood counts, pyrexia and abdominal pain syndrome were uncommon and mild and were not thoroughly recorded in this study.

## 3. Discussion

Severe cGVHD, solid organ transplantation and cutaneous T-cell lymphoma are approved indications for ECP. Any other indications, including various autoimmune disorders, have low strength of recommendation [11,14]. Probably, unique mechanism of ECP action has immunological consequence only in the three abovementioned disorders.

Less than 20% of patients with cGVHD adequately respond to initial therapy, thus presuming steroid resistance to be a common clinical situation [15]. Hence, the majority of patients with cGVHD would require treatment in addition to steroid therapy. Though mild cGVHD can be often managed with local therapies, systemic treatment is also a rational option in rare cases, according to established criteria [16]. In the present ECP study, several children with mild cGVHD did not significantly influence overall data analysis, due to low number of such patients. 

The reported data on long-term outcomes in children with SR cGVHD are limited. Therefore, emergence of new treatment strategies requires regular re-evaluation of optimal management [1]. All existing anti-GVHD drugs, or non-pharmacological methods show a significant rate of treatment failure [4,17]. Treating physician needs a wide range of therapeutic options to perform comprehensive cGVHD management. A significant proportion of children (43%) in our study also required further correction of IST after ECP, due to inadequate or transient clinical response. Hence, several equally effective treatment options should be available in order to manage cGVHD patients. In most instances, only combination therapy may provide control of SR cGVHD [18]. Another interesting and yet poorly studied question is the order of administration of immunosuppressive therapies for SR cGVHD. It seems that this order may play a substantial role in immune tolerance induction.

According to data reported elsewhere, ORR and OS in patients with SR cGVHD after ECP exceed 60% [19]. Our study demonstrated similar results, i.e., a high response rate to ECP-based therapy in all the organs, except of lungs. When discussing the response to ECP one should assume that patients always receive combination IST with ECP being only a component of therapeutic approach, so whether there is a genuine response to ECP or response to combination IST remains unanswered. Steroid-sparing effect of ECP-based therapy was demonstrated in our study with median dosage of steroids of 0.5 mg/kg prior to treatment and 0.25 mg/kg after the therapy (*p* = 0.0003). This important benefit of ECP reduces adverse effects associated with prolonged steroid therapy. Chronic GVHD in children could theoretically have some specific features compared to adults, like in other diseases. This opportunity is supported by different immune reconstitution features in children and adults [20]. But we didn’t observe any differences in response rate and survival in children with SR cGVHD compared to adults, in terms of ECP effectiveness. Similar data had been registered earlier in pediatric population [21].

Despite established criteria for cGVHD response, assessment of this important outcome measure is still inaccurate, due to subjective clinical interpretations, especially in retrospective analysis. OS is probably the most important and objective endpoint. In the present study, long-term OS (57%) and PFS (56%) were relatively high for this unfavorable group which predominantly comprised of high-risk patients. Relatively low level of CIR (23%) demonstrates fundamental opportunity of ECP to control SR cGVHD, without interfering in graft-versus-tumor effect [22,23,24]. The present study seems to be the first to demonstrate FFS in pediatric population treated with ECP. This important outcome measurement demonstrates more complex characteristics of therapy efficiency as compared to simple response to ECP. In our study, the failure-free survival rate was 30%. It means that only one-third of the children can be cured from cGVHD after ECP. The majority of patients, despite ECP, would require additional IST, due to persistence of clinically significant cGVHD symptoms. Anyway, ECP remains established and safe treatment options with only uncommon catheter-associated complications and absence of clinically significant systemic adverse effects. These data are in accordance with previously published results [25,26]. Chronic GVHD is usually characterized by low incidence of CR, despite the type of applied therapy. Therefore, various treatment modes are used in order to achieve the best response. The same patterns of response in cGVHD and classical scleroderma suggest common pathogenetic features of both conditions [27].

ECP in children is associated with several patient-specific parameters [28]. Low weight is among most significant factors that prevent classical ECP in children < 8 kg, due to their inability to tolerate this extracorporeal procedure. Low-weight patients could be managed by means of small-scale ECP technique if not eligible for apheresis [29]. Hence, ECP can be performed in any patient regardless of weight.

Some investigators demonstrated improved OS in responders to ECP compared to non-responders [30]. Such approach seems to be somewhat speculative, since the group characteristics are not specified in details and the patients from these two groups may differ in their fundamental characteristics. Moreover, treatment of non-responders to ECP has changed nowadays, due to introduction of novel effective drugs, e.g., ruxolitinib. Non-responders could be also refractory to any IST applied (not necessary ECP), for some intrinsic biological reasons not associated with treatment. This biased design strategy prevents direct comparison of the two groups differing in response to ECP.

Nevertheless, improved OS in our study was associated only with clinical response to ECP. These data support fundamental need for cGVHD control in allo-HSCT survivors. All other patient and transplant characteristics didn’t impact survival, probably, due to limited number of enrolled patients. Addition of ruxolitinib treatment to ECP also didn’t result in improved survival. These data can’t replace direct comparisons between ECP and ruxolitinib therapy, since the patients subjected to combination therapy exhibit more resistant course of cGVHD. Further studies to define most optimal cGVHD strategies and their application order are quite reasonable. In our study, ECP was safely co-administered with ruxolitinib in eight children (19%). This is in concordance with recently published data and proves a fundamental opportunity to combine these treatment options, if required [18].

Durable response is the main goal of the GVHD therapy, and this effect may be achieved only after immune tolerance induction. Generally, cGVHD prophylaxis should lead to tolerance between donor and recipient cells by ca. 3 months after allo-HSCT [7]. In case of cGVHD, tolerance cannot be achieved after standard drug prophylaxis, or this balance may be impaired. ECP results not only in cGVHD symptoms amelioration, but it also provides extra time for development of physiological tolerance. Better understanding of immune tolerance induction in patients with cGVHD is important since it would lead to the appearance of new treatment paradigms. According to prolonged FFS observed in the present study, ECP-based therapy induces continuous immune tolerance in, approximately, one-third of children with SRcGVHD. In general, even after ECP-failure, cGVHD was well controlled, i.e., only four patients in the study succumbed due to cGVHD. This fact hypothesizes that ECP can make immune system more sensitive for further therapy. Long-term NRM was 14%, comparable to previously published data [1]. This level should be regarded as high, and additional, more effective treatment options are needed for cGVHD control in non-responders to ECP. Ruxolitinib and ibrutinib are among the most promising agents in these clinical situations.

## 4. Materials and Methods

The study was performed in RM Gorbacheva Research Institute at the I.Pavlov University (St. Petersburg, Russian Federation). A total of 42 children with SR cGVHD were enrolled. Median age was 13 years (1–18). There were 25 (59%) males and 17 (41%) females in the study. The majority of patients had acute leukemia (*n* = 32, 76%), other diagnoses included myelodisplastic syndrome/juvenile myelomonocytic leukemia (MDS/JMML) (*n* = 3, 7%), Fanconi anemia (*n* = 2, 5%), chronic myelogenous leukemia (*n* = 2, 5%), Hodgkin’s disease (*n* = 2, 5%) and Hurler syndrome (*n* = 1, 2%). Patients were attributed to the high-risk group (*n* = 30, 71%) in case of acute leukemia (≥2 remissions) and MDS/JMML, all other patients were classified to standard risk group prior to allo-HSCT. GVHD prophylaxis was mostly based on the post-transplant cyclophosphamide (PtCy) (*n* = 18, 43%) and anti-thymocyte globuline (*n* = 18, 43%). HLA-matched related donors were used as stem cell source in seven patients (17%); haploidentical donors, in 17 patients (40%); HLA-matched unrelated donors, in 13 (31%), and mismatched unrelated donors, in five cases (12%). Myeloablative and reduced-intensity conditioning regimens were used in 18 (43%) and 24 (57%) patients, respectively. Transplant source was bone marrow (*n* = 30, 71%) and peripheral blood (*n* = 12, 29%).

Diagnosis and classification of cGHVD was based on established NIH criteria [16]. Mild, moderate and severe cGVHD was observed in three (7%), 15 (36%) and 24 (57%) patients, respectively. Classical cGVHD was diagnosed in 36 (86%) children and overlap syndrome in six (14%). Among children with mild cGVHD, one patient received therapy due to non-malignant disease and awareness of cGVHD progression. In two other cases, systemic immunosuppressive therapy (IST) was initiated by decision of attending physician. Several organs were affected in 32 (76%) patients and only one organ, in 10 cases (24%). In a majority of patients, skin (*n* = 32, 76%), mucous membranes (*n* = 22, 52%), liver (*n* = 10, 24%) and lungs (*n* = 9, 21%) were involved. Less common presentations included gut (*n* = 5, 12%) and joints (*n* = 3, 7%).

First line therapy of cGVHD was methylprednisolone 1 mg/kg/day in all patients with further gradual tapering according to response and tolerance. Criteria for SR cGVHD were progression despite this approach for 1–2 weeks, stable disease while on 0.5 ≥ mg/kg/day for 1 month or inability to withdraw steroids without flare of the disease. Four patients (10%) had intolerance to steroids due to unacceptable toxicity (infectious or metabolic complications). SR cGVHD was diagnosed in 38 children (90%). ECP was administered in patients on individual basis after discussion on board of doctors.

All the patients received ECP as second (*n* = 18, 43%) or third (*n* = 24, 57%) line of therapy. Initial ECP therapy consisted of bimonthly treatment schedule for two consecutive days with an opportunity of further tapering, according to clinical response. Any concurrent treatment administered before ECP could be continued if considered necessary. ECP was co-administered with ruxolitinib in eight (19%) children, sirolimus in five (12%), imatinib in six (14%), rituximab in two (5%) and ibrutinib in one (2%).Detailed characteristic of concomitant IST and requirement for systemic therapy escaltion after ECP is shown in Table 1. All the patients received off-line ECP procedures with a MacoGenic device (Macopharma, Lille, France) according to the standard protocol [31]. Apheresis was performed with a Cobe Spectra device (Terumo, Lakewood, CA, USA). Sodium citrate was the anticoagulant used during mononuclear cell collection. The final volume of the product was 150 mL. It was further diluted with normal saline up to 300 mL. Afterwards a photosensitizing agent (8-methoxypsoralen) was added to the product up to concentration of 200 ng/mL with subsequent ultraviolet irradiation (320–400 nm) of 2 J/cm^2^. Total duration of the procedure was approximately 3 h.Median number of ECP procedures was 10 (2–46), with total number of 606, and median duration for 4 months (1–42). Median interval from allo-HSCT to first ECP was 7 months (4–41). Clinical response to ECP-based therapy was evaluated by means of established criteria [32]. Complete response was diagnosed in patients with resolution of all cGVHD symptoms. Partial response (PR) was diagnosed in case of improvement in ≥1 organ or site without any signs of deterioration in other localizations. PR was diagnosed after decrease in clinician overall severity score by ≥2 points according to the 2014 Response Criteria Working Group Report. Progression of cGVHD was defined separately for each organ based on the above mentioned recommendations. Patients that did not meet criteria of CR, PR or progression were classified as unchanged. If response to ECP-based therapy was registered than patients were gradually weaned away from steroids at a rate of 25% dose reduction biweekly. Simultaneously with steroid tapering the ECP schedule was reevaluated every 3 months with the switch to monthly or quarterly procedures in responders on an individual basis. Overall survival (OS), progression-free survival (PFS) and failure-free survival (FFS) were calculated by Kaplan-Meier method. Failure-free survival was defined as the absence of relapse, non-relapse mortality or addition of another systemic therapy. Events for FFS were considered relapse, death, or IST escalation. Non-relapse mortality (NRM) and cumulative incidence of relapse (CIR) were assessed using competing risks. Univariate analysis was performed by Cox regression. The Mann-Whitney U test was used to compare whether there was difference in the dependent variable for two independent groups. Variables related to patient, disease, and transplants were characterized using descriptive statistics. For statistical analysis we used the Easy R (EZR) program [33].

## 5. Conclusions

ECP remains an important treatment option in children with SR cGVHD, despite emergence of new effective options, due to safety and high ORR, with similar clinical efficiency in pediatric and adult patients. To our knowledge this is probably the first publication demonstrating FFS in children with cGVHD after ECP. Suboptimal FFS represents potential of ECP-based therapy to cure only third of children with cGVHD. A significant proportion of patients would require escalation of IST to control cGVHD. This means that ECP should not be regarded as a game changer but as an important approach that can help to relieve symptoms in most of the patients at least transiently. 

## Figures and Tables

**Table 1:** Demographics of patients with cGVHD treated with ECP.

№	Age	Diagnosis	cGVHD Severity	Affected Organs	Steroids Prior ECP, mg/kg/day	Number of ECP Procedures	Response to ECP	Steroids Post ECP, mg/kg/day	Concomitant IST	Requirement for IST Escalation after ECP	Status (Cause)	Follow-Up, Days
1	13	MDS	severe	skin, mucous membranes	0.5	8	CR	0.1	no	no	alive	1466
2	5	AML	moderate	skin	0.4	5	CR	0	CsA	no	alive	4541
3	16	ALL	moderate	skin, lungs	0	12	PR	0	CsA	no	alive	4064
4	10	ALL	severe	skin	1	7	progression	1	Tx	yes	dead (ALL)	180
5	10	ALL	moderate	skin	0.5	4	progression	2	ruxolitinib	yes	alive	721
6	18	ALL	severe	skin, mucous membranes	0.3	4	PR	0.3	ibrutinib	no	alive	463
7	10	BAL	moderate	skin, mucous membranes, lungs	0.5	8	PR	0.2	no	no	dead (ALL)	1097
8	13	ALL	moderate	joints, gut	0.6	6	PR	0.25	CsA	no	alive	774
9	5	AML	moderate	mucous membranes	0.5		PR	0.5	no	no	alive	256
10	13	BAL	severe	liver	0.3	10	PR	0	sirolimus	no	dead (BAL)	396
11	8	CML	severe	mucous membranes	1	24	PR	0.25	imatinib	no	alive	3929
12	12	AML	severe	skin, liver, lungs	0.7	20	CR	0.1	imatinib	yes	alive	1834
13	1	ALL	severe	skin, lungs	0.25	2	PR	0.1	CsA	no	alive	3394
14	13	ALL	moderate	skin, liver	0.4	2	PR	0.4	ruxolitinib	no	dead (ALL)	352
15	3	Hurler	mild	skin, mucous membranes	0.5	6	PR	0.2	ruxolitinib	yes	alive	864
16	5	AML	moderate	skin, gut	0.5	26	PR	0.3	imatinib	no	alive	1217
17	16	AML	severe	skin, mucous membranes, liver, lungs	0.4	33	unchanged	0.75	rituximab	yes	dead (cGVHD)	604
18	18	AML	severe	mucous membranes, liver	0.6	12	PR	0.25	Tx	no	alive	3683
19	13	ALL	mild	skin, mucous membranes	1	5	PR	0.5	sirolimus	yes	dead (cGVHD)	201
20	16	AML	mild	skin, liver	0.8	28	PR	0	Tx	no	alive	1379
21	4	Fanconi	severe	skin, gut	0.5	6	PR	0.25	Tx	no	alive	3140
22	11	ALL	severe	skin	0.4	24	CR	0.1	ruxolitinib	no	alive	1334
23	14	Fanconi	severe	skin, joints, lungs	0.6	12	PR	0.4	ruxolitinib	no	dead (Fanconi)	259
24	1	AML	severe	skin, mucous membranes, liver	0.25	10	PR	0.1	ruxolitinib	no	alive	1453
25	8	ALL	moderate	skin, gut, liver	0.7	18	PR	0.25	Tx, mmf	no	dead (ALL)	182
26	15	AML	severe	skin, mucous membranes, lungs	0.4	2	progression	0.5	Tx	yes	dead (cGVHD)	249
27	7	ALL	moderate	mucous membranes	0.6	37	PR	0.8	sirolimus	yes	alive	648
28	6	ALL	severe	skin, mucous membranes, lungs	0	35	unchanged	0	rituximab	yes	dead (cGVHD)	587
29	6	ALL	severe	skin, mucous membranes, gut, liver	0	40	PR	0	imatinib	yes	alive	1246
30	3	BAL	moderate	skin, mucous membranes	0.4	4	CR	0	Tx	no	alive	4167
31	17	BAL	severe	mucous membranes	0.3	10	progression	0	imatinib	yes	dead (BAL)	325
32	13	ALL	moderate	mucous membranes	0.5	12	PR	0.25	sirolimus	no	alive	342
33	15	ALL	moderate	skin	1	34	CR	0.1		no	alive	400
34	7	JMML	moderate	skin	0.25	5	CR	0.1	Tx	no	alive	2800
35	16	HL	severe	mucous membranes	0.4	14	PR	0.25	ruxolitinib	yes	alive	590
36	17	ALL	severe	mucous membranes	0.7	5	progression	0.7	CsA	yes	dead (ALL)	252
37	16	CML	severe	skin, joints	0.3	34	PR	0.3	CsA	yes	alive	3568
38	17	HL	severe	skin, mucous membranes, lungs	0.5	46	unchanged	0.5	ruxolitinib	yes	alive	536
39	9	JMLL	moderate	skin, mucous membranes	0.4	42	PR	0.25	sirolimus	no	alive	2146
40	16	AML	severe	skin, mucous membranes	0	10	progression	0	imatinib, Tx	yes	dead (AML)	870
41	18	ALL	severe	skin	0.4	8	progression	0	Tx, mmf	yes	dead (ALL)	270
42	17	AML	severe	skin, liver	0.5	8	progression	0.75	no	yes	dead (cGVHD)	239

MDS—myelodysplastic syndrome, AML—acute myeloid leukemia, ALL—acute lymphoblastic leukemia, BAL—biphenotypic acute leukemia, JMML—juvenile meylomonocytic leukemia, HL—Hodgkin lymphoma, CML—chronic myelogenous leukemia, ECP—extracorporeal photopheresis, CR—complete response, PR—partial response, IST—immunosupressive therapy, CsA—cyclosporine A, Tx—tacrolimus, mmf—mycophenolate mofetil, cGVHD—chronic graft versus host disease.

**Table 2:** Clinical response to extracorporeal photophersis in children with cGVHD.

Efficiency of ECP in Children with cGVHD							
Response	Skin (*n* = 32)	Mucous Membrane (*n* = 22)	Lungs (*n* = 9)	Liver (*n* = 10)	Gut (*n* = 5)	Joints (*n* = 3)	Overall Response (*n* = 42)
Complete response, *n* (%)	7 (22%)	2 (9%)	1 (11%)	2 (20%)	N/A	N/A	7 (17%)
Partial response, *n* (%)	17 (53%)	14 (64%)	1 (11%)	6 (60%)	4 (80%)	2 (67%)	24 (57%)
Unchanged, *n* (%)	4 (12.5%)	3 (13.5%)	6 (67%)	1 (10%)	1 (20%)	1 (33%)	3 (7%)
Progression, *n* (%)	4 (12.5%)	3 (13.5%)	1 (11%)	1 (10%)	N/A	N/A	8 (19%)

N/A—not applicable.

**Table 3:** An univariate analysis of factors influencing survival.

Variable	HR	95%CI	*p*
Response to ECP	7.8	2.7–23.1	0.0002
Sex	2.3	0.6–8.7	0.2
Disease risk group (HR vs. SR)	1.964	9–0.4	0.4
Time interval from HSCT to ECP	0.4	0.1–1.5	0.2
Ruxolitinib− vs. Ruxolitinib+	1.7	0.5–6.9	0.4
ECP monotherapy vs. ECP in combination therapy	0.6	0.2–2.4	0.5
cGVHD severity (severe vs. moderate)	2.656	0.8–8.3	0.1

ECP—extracorporeal photopheresis, HR—high risk, SR—standard risk, HSCT—hematopoietic stem cell transplantation, cGVHD—chronic graft versus host disease, ruxolitinib− (absence of ruxolitinib in combination therapy), ruxolitinib+ (presence of ruxolitinib in combination therapy).

## Data Availability

The data are not publicly available due to privacy and ethical restrictions.
